# Distinct pathways of solid-to-solid phase transitions induced by defects: the case of dl-me­thio­nine

**DOI:** 10.1107/S2052252521004401

**Published:** 2021-05-08

**Authors:** Genpei Shi, Si Li, Peng Shi, Junbo Gong, Mingtao Zhang, Weiwei Tang

**Affiliations:** a School of Chemical Engineering and Technology, State Key Laboratory of Chemical Engineering, Tianjin University, 300072, Tianjin 300072, People’s Republic of China; b The Co-Innovation Center of Chemistry and Chemical Engineering of Tianjin, Tianjin 300072, People’s Republic of China; c College of Chemistry, Nankai University, Tianjin 300071, People’s Republic of China

**Keywords:** polymorphs, solid-to-solid phase transitions, crystal defects, cooperative molecular motion, nucleation and growth, polymorphism, pharmaceutical solids

## Abstract

Understanding of solid-to-solid phase transition mechanisms in polymorphic systems is of critical importance for rigorous control over polymorph purity in the pharmaceutical industry to achieve the desired bioavailability and efficacy of drugs. Ubiquitous defects in crystals may play an important role in the pathways of phase transitions. However, such effects remain poorly understood. Here, the effects of crystal defects on the solid-to-solid phase transformations between dl-me­thio­nine polymorphs α and β are investigated by means of experimental and computational approaches. Thermal analyses of polycrystalline powders show two endothermic peaks in the α-to-β phase transition (and two exothermic peaks for the reverse transition), in contrast with one thermal event observed for single crystals. Variable-temperature 1D and 2D Raman spectra, as well as powder X-ray diffraction patterns, reveal the appearance of two peaks that can attributed to a two-step phase transition, and the extent of the second-step phase transition increases with milling time (or defect density). Quantification of transition kinetics unveils a remarkably higher energy barrier in the second-step phase transition than in the first, proceeding by the cooperative molecular motion pathway. The good linear fitting on the kinetic data by the Jeziorny model suggests that the second-step transition follows the nucleation and growth mechanism. Molecular dynamics simulations were also conducted to understand the role of crystal defects in the solid-state phase transition by tracking the atomic distribution and hydrogen bond lifetime during the transition. It was found that the increasing defect density hinders the propagation of cooperative molecular motion, leading to a combined transition mechanism involving both cooperative motion and nucleation and growth. This study highlights the significant impact of crystal defects on solid-state phase transitions, and the two-step transition mechanism postulated may be universal given the ubiquitous presence of defects in crystalline materials.

## Abstract 2

Ubiquitous defects in polycrystalline powders and single crystals affect the pathways of polymorphic solid-to-solid phase transitions. Dependent on the density of crystal defects, the phase transition mechanism can be dominated by either the cooperative molecular motion pathway or the nucleation and growth pathway.

## 1. Introduction   

Polymorphs of crystal structures often display distinct physical properties such as solubility (Williams *et al.*, 2013 ▸), dissolution rate (Blagden *et al.*, 2007 ▸) and elastic modulus (Bernstein, 2010 ▸) which are of significant importance for the manufacturing of pharmaceuticals and drug efficacy (Bauer *et al.*, 2001 ▸; Singhal & Curatolo, 2004 ▸). Thermodynamic stability among polymorphs is dependent on their Gibbs energy difference, and phase transformation from one form to another is driven by a solvent-mediated or solid-state phase transition. In contrast, with dissolution and recrystallization from solution by solvent-mediated phase transformation (Greco & Bogner, 2012 ▸), solid-state phase transitions from the metastable to stable form in the solid state are limited by atomic movement and diffusion (Herbstein, 2006 ▸). Importantly, solid-state phase transitions of pharmaceutical crystals will reduce the shelf-life and the bioavailability of a drug. Understanding of such transition mechanisms is beneficial to control the polymorphic forms in all development stages of pharmaceuticals.

Solid-to-solid phase transitions in organic systems are often achieved by the nucleation and growth mechanism (Peng *et al.*, 2015 ▸; Krishnan & Sureshan, 2015 ▸), consisting of two steps: nucleation and growth of a new crystal phase. There exists an appreciable interface between the old crystal phase and the new one, and atoms in the parent (old) phase will cross the interface individually and disorderly to assemble into the new crystal phase. Nonetheless, recent studies found a cooperative single-crystal-to-single-crystal phase transition mechanism in a number of polymorphic systems displaying similar crystal structures by collective and rapid propagation of molecules (Chung *et al.*, 2018 ▸; Herbstein, 2006 ▸). A solid-state phase transition mechanism of this nature is usually observed when polymorphs respond to external light or thermal stimuli in which crystals often display mechanical movements such as translation, rotation, jumping, bending and torsion (Reddy *et al.*, 2010 ▸; Sahoo *et al.*, 2013 ▸; Karothu *et al.*, 2016 ▸; Liu *et al.*, 2017 ▸; Commins *et al.*, 2016 ▸).

If proceeding by atomic displacement or diffusion, a solid-state phase transition may be significantly influenced by crystal defects which are ubiquitous in crystals. Various types of defects were reported in crystals such as (sub-)grain boundaries, dislocations and vacancies, leading to the appearance of lattice distortion and high distortion energy (Beyerlein *et al.*, 2015 ▸). It was found that the large fluctuation of energy, structure and composition within crystal defects often promotes the nucleation of a new phase (Mnyukh, 2010 ▸). The solid-to-solid phase transition at (or near) regions of crystal defects demonstrated high diffusion velocity and fast transition stress relaxation because of its low atomic diffusion activation energy (Smets *et al.*, 2020 ▸). More recently, it was suggested that the strain produced during the cooperative single-crystal-to-single-crystal phase transition may be dissipated at the defect sites, reducing the accumulation of energy for cooperative molecular displacement (Naumov *et al.*, 2015 ▸). By this action, defects will interrupt the propagation of a new phase in the crystal layers (Smets *et al.*, 2015 ▸). However, the effects of crystal defects on polymorphic phase transitions in polycrystalline powders were not well understood and, more importantly, the dual role of crystal defects in both nucleation-growth and cooperative transition mechanisms has not, to the best of our knowledge, been explored.

**Figure 1:**
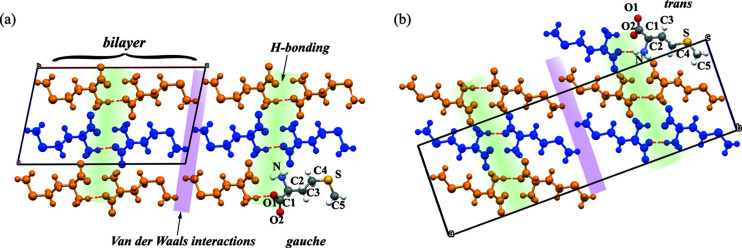
Schematic of DL-MET crystal structures of the (*a*) α polymorph (CSD entry DLMETA07, only the major conformation is displayed) and (*b*) β polymorph (CSD entry DLMETA09) viewed along the *b* axis.

Herein, we explore the effects of crystal defects on solid-state polymorphic phase transitions using dl-me­thio­nine as a model compound. dl-Me­thio­nine is the only sulfur-containing essential amino acid. Two polymorphs, α and β, of dl-me­thio­nine have been reported and are part of an enantiotropic system whereby one polymorph can transform to the other reversibly (Groom *et al.*, 2016 ▸). The β polymorph is the thermodynamically stable form at room temperature, whereas the α polymorph is more stable above the transition temperature, reported to be 306–325 K (Lesage *et al.*, 1999 ▸; Kawakami, 2007 ▸). Both crystal structures consist of hydrogen-bonded bilayers with van der Waals interactions between the bilayers. The main difference between the two polymorphs lies in the stacking sequence of bilayers and the conformation of the molecular side chains (*trans* versus *gauche*; Fig. 1 ▸) (Taniguchi *et al.*, 1980 ▸). Previously, Smets found that cooperative motion plays a role in the solid-state phase transition between α and β and the kinetic energy barrier of the phase transition in polycrystalline powders is greater than for single crystals (Görbitz & Karen, 2015 ▸; Smets *et al.*, 2016 ▸). Crystal quality was postulated as a contributing factor to the transition barrier in polycrystalline powders, but details of the effects such as macroscopic size and crystal quality as well as microscopic crystal defects on phase transitions between polymorphs have not been resolved. In this contribution, we study how the presence of crystal defects plays a dual role in both cooperative molecular motion and distinct nucleation and growth transition pathways.

## 2. Experimental and computational details   

### 2.1. Material and polymorph preparation   

dl-Me­thio­nine (≥99.0%) was purchased from Tokyo chemical industry Co. Ltd (TCI) and deionized water was used to prepare the solution. dl-Me­thio­nine solution of 15–30 mg ml^−1^ was prepared by dissolving known amounts of crystals in water at 323 K, which was then filtered through 0.22 µm membrane filters. The filtrate was used to grow β single crystals by a series of crystallization methods including cooling, slow evaporation and hanging drop crystallization to obtain better quality single crystals. For a typical single crystal grown by cooling crystallization, the filtrate was rapidly cooled to 277 K and kept for 3–15 days at this temperature, yielding single crystals about 400 µm in size. Larger single crystals (600–1300 µm) were obtained from slow evaporation. A 5 ml saturated aqueous solution of dl-me­thio­nine at 303 K was prepared, filtered with a 0.22 µm water-based filter membrane and transferred to a small clean beaker for slow evaporation of solvents. Single crystals ranging from 30 to 200 µm were obtained by rapid evaporation crystallization. A 10 µl filtrate was dropped onto a clean glass substrate (1 × 1 cm) and left in a hood for spontaneous evaporation at room temperature and moderate humidity for 1–2 h. All polarized microscopic images of the single crystals prepared were collected on a Zeiss Primotech microscope (Germany) and the sizes were measured. All the prepared crystals were treated gently to protect the crystal integrity. Different quality samples were examined using scanning electron microscopy (Hitachi S-4800) (Fig. S1 of the supporting information).

Polycrystalline α powders were prepared by directly heating dl-me­thio­nine raw materials (crystal mixture of α and β forms) at 353 K for 48 h in the oven, and β polycrystalline powders were prepared by cooling crystallization. A total of 16 g of dl-me­thio­nine was dissolved in 200 ml deionized water with a stirring rate of 300 rpm at 333 K. When the crystals were fully dissolved, the clear solution was firstly cooled to 293 K at a rate of 20 K min^−1^, then to 283 K at a rate of 10 K min^−1^ and kept at 283 K for 48 h. Different cooling rates were applied to reduce the overall crystallization time and ensure the full transformation of any α crystals.

### 2.2. Mechanochemical milling   

Ball milling using a Mixer Mill MM400 (Retsch GmbH, Haan, Germany) was employed to reduce the size of crystals and generate more crystal defects. The steel ball has a diameter of 7 mm. Single crystals of the β polymorph were milled at a frequency of 10 Hz for 20, 30, 60, 90 and 180 min, respectively. Mechanical milling at excessively high frequency can lead to crystal transformation (Wang, 2013 ▸). The SEM images show macro-steps at the surface of the powder crystallite in Fig. S1. Milling may increase the mosaicity of the crystals, generating a domain structure.

### 2.3. Thermal analysis   

Differential scanning calorimetry (DSC) measurements were performed using the Mettler–Toledo DSC1/500 calorimeter with a high-sensitivity sensor (HSS8). The crimped aluminium pans loaded with 5–10 mg samples (either single crystals or polycrystalline powders) were heated at temperatures ranging from 223 K to 400 K under nitro­gen protection. A heating/cooling rate of 5 K min^−1^ was used throughout all the measurements.

### 2.4. Raman spectroscopy   

Variable-temperature (VT) Raman spectroscopy was employed to monitor the transition of crystal forms during the cycle of heating and cooling. Spectra were collected on a DXR Microscope (USA) equipped with an He–Ne laser at 532 nm and a thermoelectrically cooled CCD detector. At least ten scans were acquired for each sample from 0 to 3500 cm^−1^ at a resolution of 1.2 cm^−1^ with an exposure time of 5 s. The sample was heated at 5 K min^−1^ from 223 to 393 K and then cooled at the same rate down to 223 K. Raman spectra were collected at respective temperatures of 223, 318, 343, 363, 393, 363, 333, 303, 253 and 223 K.

Spatially resolved 2D Raman spectroscopy was employed to understand the partial transition behaviour of dl-me­thio­nine α and β forms. Spectra were collected on an *InVia* Reflex Raman microscope (RENISHAW, UK) equipped with a 532 nm laser source and a thermoelectrically cooled CCD detector. The measurements were performed on a 60 × 70 µm area with a scan step interval of 10 µm. Each spectrum was acquired in the range 600–1800 cm^–1^. The heating and cooling procedures were the same as those of the above mentioned 1D VT-Raman spectroscopy process. Raman spectra of the samples were collected at respective temperatures of 223, 343, 363, 393, 333 and 253 K. 2D Raman maps were generated using an intensity-based colour scale. The characteristic peaks of α and β are 762 and 782 cm^−1^, respectively. The relative intensity value of the peak at 762 cm^−1^ over the peak at 782 cm^−1^ was used to track the evolution of polymorphic transformation.

### 2.5. Powder X-ray diffraction   

Powder X-ray diffraction (PXRD) data were obtained on a Rigaku D/MAX 2500 X-ray diffractometer (Rigaku, Japan) utilizing Cu *K*α radiation (λ = 1.54178 Å) at 100 mA and 40 kV. Data were acquired at 298 K to confirm the crystal forms of dl-me­thio­nine. The range of the scanning 2θ angle was 10–50° at a speed of 8° min^−1^. The remaining powders after 180 min milling were found to be the β form, confirmed from the PXRD pattern. VT PXRD data were acquired at 298–373 K to observe crystal changes *in situ*. Samples were scanned in the 2θ range 10–40°.

### 2.6. Hot-stage polarized optical microscopy   

Polycrystalline powder samples milled for 30 min were placed in a heating chamber under a polarized light microscope (PLM, BX51 with polarizer filter, Olympus Optical Co. Ltd, Japan) equipped with a hot-stage platform (LTS350, Linkam Scientific Instrument, Ltd, UK). The heating chamber was capped with a sealable lid during heating and cooling temperature cycles. The phase transition experiments were performed in the temperature region between 293 and 383 K at a rate of 5 K min^−1^. The stability of the controlled temperature was ± 0.1 K.

### 2.7. Molecular dynamics simulations   

A molecular dynamics (MD) simulation was used to evaluate the potential impact of crystal defects on the solid-to-solid phase transition between the α and β forms. Two temperatures of 223 and 400 K were chosen, and crystal defects of different vacancy densities were created in one surface of one of the bilayers. The simulations were carried out on periodic systems of both α (4 × 10 × 5 unit cells) and β (5 × 10 × 2 unit cells) supercells with the *GROMACS* software (version 2018.4; Van Der Spoel *et al.*, 2005 ▸) using the Amber99sb-ildn force field. Partial atomic restrained electrostatic potential (RESP) (Cornell *et al.*, 1993 ▸) charges were produced from the optimized geometrical structures at the M06-2x/6-311++g(2d,2p) level using the *GAUSSIAN* software (Frisch *et al.*, 2009 ▸). Simulations were performed in the isobaric isothermic ensemble (NPT) at various temperatures and at 1.01325 bar pressure. Temperatures were held constant using a v-rescale thermostat (Bussi *et al.*, 2007 ▸) and pressure was maintained using the Parrinello–Rahman MD (Parrinello & Rahman, 1982 ▸). Electrostatic calculations were performed using the particle-mesh Ewald (PME) method with 0.9 Å grid spacing and a pseudo-2D summation. The time step was set at 2 fs and the cutoff distances of Coulomb interaction and Lennard–Jones interactions were both 9 Å. After energy minimization, the β polymorph was heated from 223 to 400 K in 10 ns and then simulated for 240 ns at 400 K, and the α polymorph was simulated in 10 ns from 400 to 223 K.

## 3. Results and discussion   

### 3.1. Differential scanning calorimetry   

#### 3.1.1. Single-crystal versus powder DSC   

Single crystals of the dl-me­thio­nine β polymorph were prepared by evaporative crystallization and further confirmed by PXRD. The β polymorph is stable under ambient conditions, but becomes metastable at elevated temperatures. DSC analyses show the onset transition temperature from the β to the α polymorph is 322–330 K in polycrystalline powders, however, it lies in 324–326 K for single crystals (Fig. S2). During the cycle of temperature cooling, the onset temperatures of the reverse transition from α to β are 295–300 K for polycrystalline powders and 301–306 K for single crystals. Single crystals generally have a narrower temperature range. Our measured onset transition temperatures of both β to α and α to β are consistent with the reported values, though with narrower ranges (Smets *et al.*, 2016 ▸). Furthermore, the large variations in the onset transition temperatures demonstrate two reversible transitions between the α and β forms and a large kinetic hysteresis.

**Figure 2:**
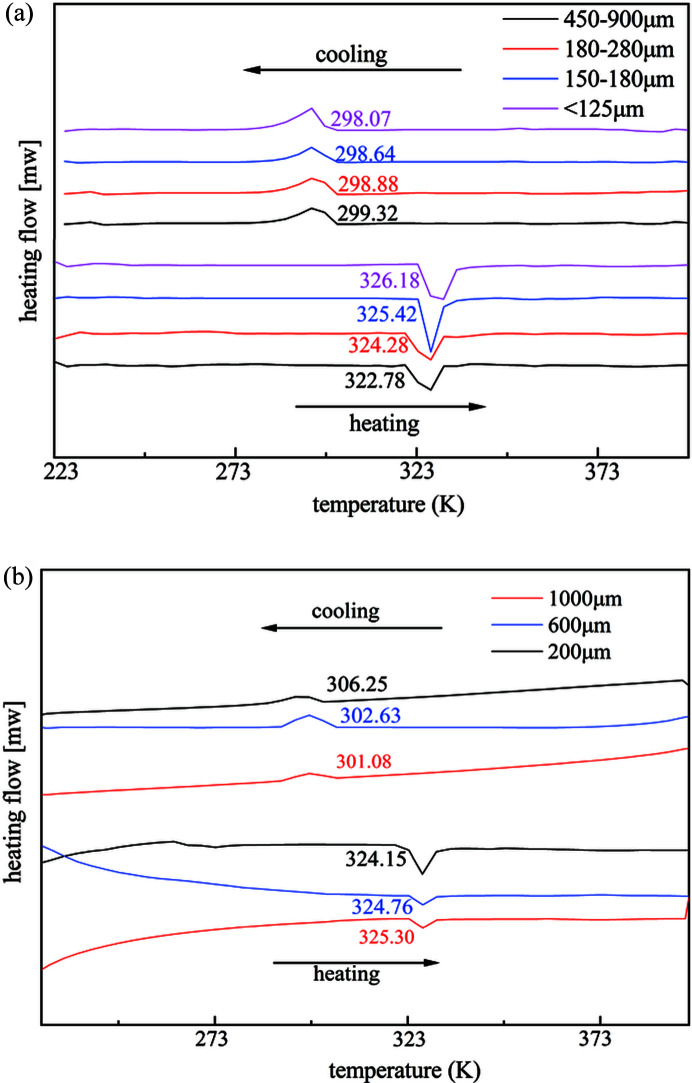
DSC thermograms of heating and cooling cycles of (*a*) polycrystalline powders of different sizes in the same batch samples and of (*b*) single crystals of different sizes.

In order to understand the variation in the onset temperature of the two reversible phase transitions observed in both single crystals and polycrystalline powders, we examined the effects of crystal size and defects. The polycrystalline powders and single crystals of the β polymorph were sieved into different size ranges and observed by polarized optical microscopy (Figs. S3 and S4). Then DSC analyses were performed for the different size categories. Fig. 2 ▸ presents DSC thermograms of heating and cooling recycling of powder and single crystals with different crystal sizes. We found that both powder and single crystals of small size display higher transition temperatures for the β-to-α transition and a lower transition point for the reverse α-to-β transition than those for larger crystals. The onset transition temperature varies from 322.78 to 326.18 K in the β-to-α transition and from 299.32 to 298.07 K in the α-to-β transition when the size of the polycrystalline powder deceases from 900 to 125 µm. For single crystals, the onset β-to-α transition temperature increases from 324.15 to 325.30 K when the crystal size is reduced from 1000 to 200 µm, whereas the α-to-β transition point significantly decreases from 306.25 to 301.08 K (Fig. S4). These observations are expected when smaller crystals present fewer defects than those of large ones.

**Figure 3:**
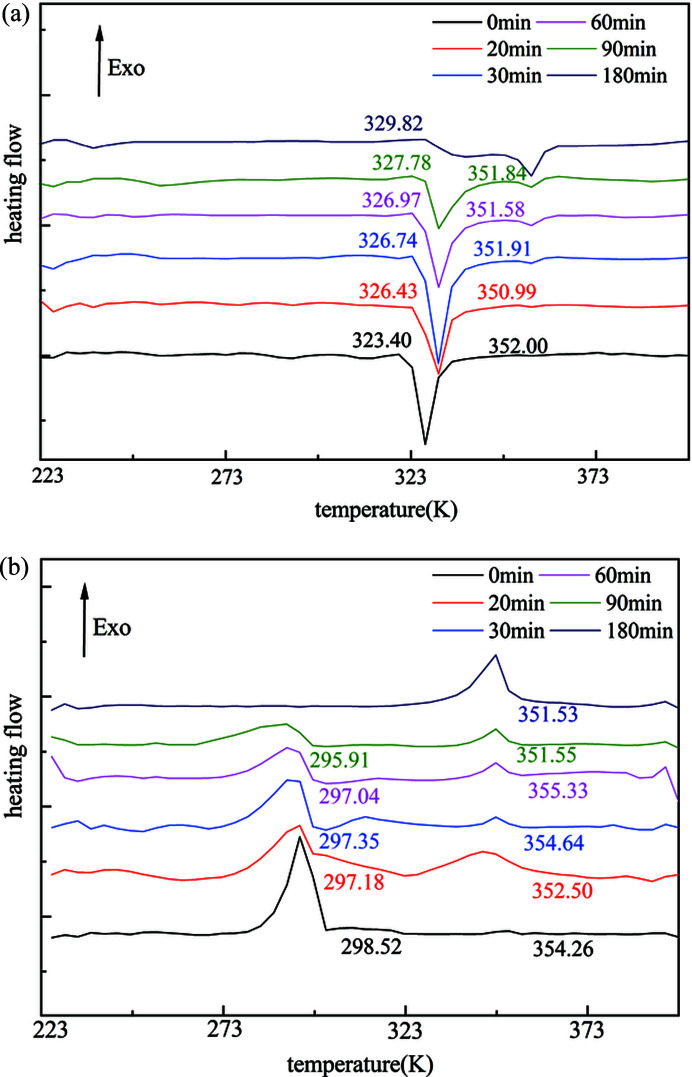
DSC temperature cycle thermograms of dl-me­thio­nine β polycrystalline powders with different milling times during (*a*) the heating process and (*b*) the cooling process.

We further examine the impact of defects on the two reversible phase transitions between α and β by intentionally inducing defects by the ball milling process. In principle, the strong ball milling process will fracture crystals and produce more defects such as domain wall and dislocations. The longer the milling time the higher the probability of generating more crystal defects. Note that during the milling process the pure β polymorph will not introduce a new phase as confirmed by PXRD and DSC thermograms (Figs. S5 and S7). Fig. 3 ▸ illustrates the DSC thermograms of the temperature recycle of milled polycrystalline powders at different time periods. The onset transition temperature of the β-to-α phase transition was found to increase remarkably from 323.40 to 329.82 K and its peak intensity decreases as the milling time increases. This is likely due to the size reduction, as observed by polarized optical microscopy (Fig. S6). More interestingly, we found that another endothermic peak around 351 K becomes significant and the onset transition temperature gradually decreases from 352.00 to 351.84 K along with the peak intensity climb as the milling time elevates. By using *in situ* VT Raman spectroscopy, we found that the appearance of the second endothermic transition peak can also be attributed to the phase transition from the β to the α polymorph but by a different transition mechanism (see the sections 3.2 and 3.3 below). It appears that the second phase transition is more sensitive to the increasing amounts of crystal defects. The wider width of the second transition peak than the first suggests a slower transition rate and a higher activation energy. In the cooling cycle, the α-to-β transition temperature along with the peak intensity were found to decreases significantly from 298.52 to 295.91 K with the increase in milling time. Again, this is probably due to the reduction of crystal size caused by ball milling. Moreover, another exothermic peak around 353 K appears, and the onset temperature of this peak varies from 354.26 to 351.53 K and displays a general decline as the milling time increases. Importantly, the peak intensity becomes significant and eventually dominant at 180 min milling time. *In situ* VT Raman spectra in the cooling cycle reveals that the second exothermic peak can be attributed to the α-to-β phase transition (see Section 3.2). The presence of the second α-to-β transition peak suggests a different transition mechanism from the exothermic peak at around 298 K. The intensity of the second transition peak gradually increases with the decline of the first transition peak (*i.e.* at 298 K), which indicates this transition process is more sensitive to the presence of defects. The wider width of the second transition peak indicates a high activation energy of the α-to-β transition.

### 3.2. Raman spectroscopy   

#### 3.2.1. Variable-temperature Raman spectroscopy   

**Figure 4:**
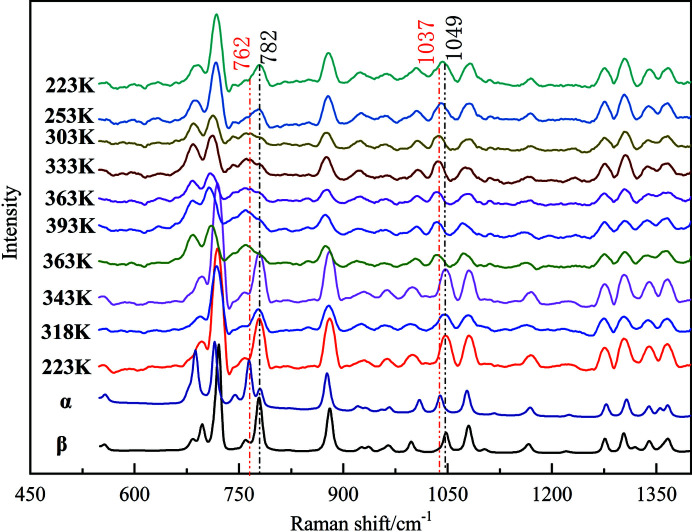
VT Raman spectra of polycrystalline powders after 90 min of milling. The temperature ranges are set from 223 to 393 K and from 393 to 223 K at a rate of 5 K min^−1^. The marked peaks are those characteristic of the β and α forms.

VT Raman spectroscopy was used *in situ* to track the structural changes throughout the solid-to-solid phase transition between the α and β forms in the temperature cycle of heating and cooling. Fig. 4 ▸ shows Raman spectra of dl-me­thio­nine crystals with varying temperature along with those of pure α and β forms. The two pure forms display spectral distinctions in the ranges 790–630 and 1030–1050 cm^−1^, which correspond to the stretching vibration of C—S and C—N bonds, respectively, owing to their conformation difference in the crystal structures. The characteristic peaks of the α polymorph are located at 762 and 1037 cm^−1^, whereas those of the β polymorph are 782 and 1049 cm^−1^. Polycrystalline powders after milling for 90 min were initially cooled to 223 K to assure pure β form, and then heated to 393 K at a rate of 5 K min^−1^ to trigger the phase transition from β to α. The crystals are β polymorph below 318 K and then undergo a phase transition between 323 and 363 K. Thus, the crystal mixtures of β and α (mainly β form) are obtained at 343 K, which suggest that the β-to-α phase transition at the first endothermic peak occurs only partially. As the temperature rises, the samples completely transform into pure α at 363 K and keep this form until 393 K. VT-Raman spectra thus support that the two transition peaks observed in DSC plots can be attributed to a two-step transition process from β to α. This conclusion is consistent with the results of VT XRD (Fig. S8). In the cooling cycle, the α-to-β phase transition starts at about 353 K and produces mixtures of the two forms at 333 K (mainly α), supporting the assignment of the second transition peak being the α-to-β phase transition. A rapid transition will occur below 298 K but not complete even after 253 K.

#### 3.2.2. Spatially resolved 2D Raman spectroscopy   

**Figure 5:**
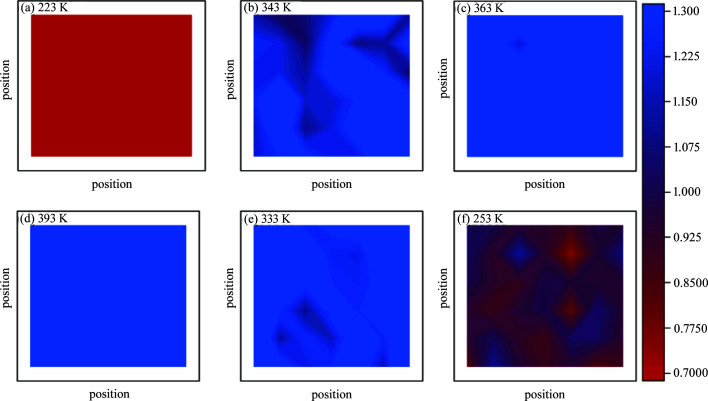
*In situ* 2D Raman mapping of polycrystalline powders after 90 min of milling (β → α → β). The horizontal and vertical coordinates represent the position of the micro-region characterized by Raman spectra, and the scale is the ratio of the peak intensity between 782 and 762 cm^−1^. Red and blue represent the pure β and α forms, respectively.

**Figure 6:**
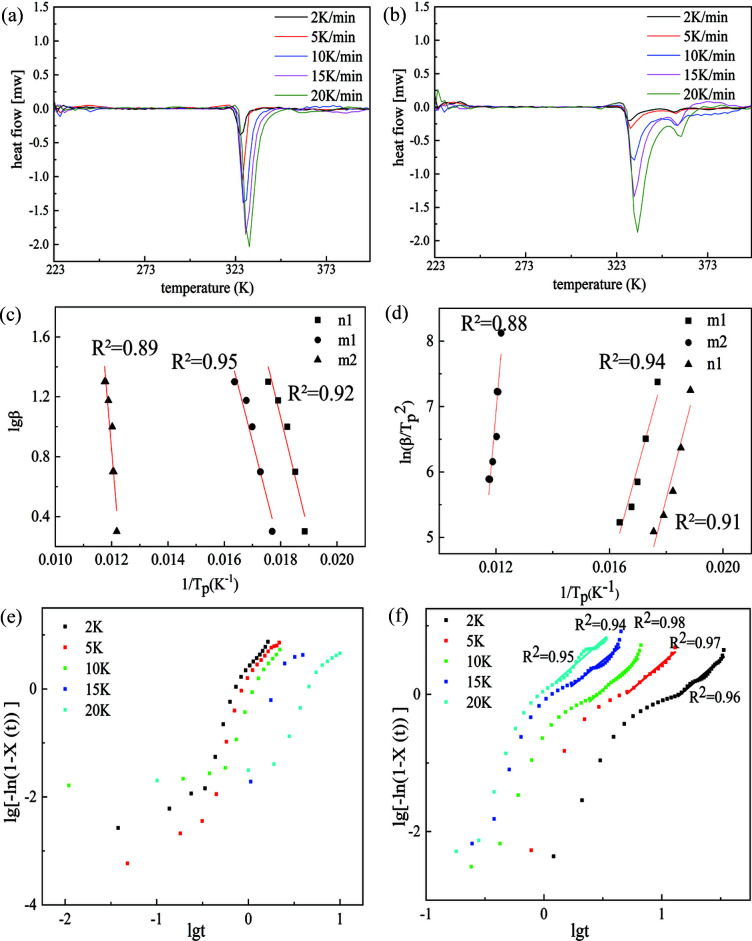
DSC thermograms of β powder crystals after (*a*) 0 min of milling and (*b*) 90 min of milling at different heating rates. Estimation of the apparent activation energy by (*c*) the Ozawa model and (*d*) the Kissinger model. n1: the first phase transition of non-milled β powders; m1 and m2: the first and second phase transitions of β powder crystals after 90 min of milling. The β → α phase transition process described by the Jeziorny model for (*e*) the non-milled polycrystalline powders and (*f*) polycrystalline powders after 90 min of milling.

To visualize the phase transition process between the α and β forms, 2D Raman mapping was applied to quantitatively analyse the polymorphic composition as it varyies with the temperature cycle of heating and cooling. The proportions of the two crystal forms were measured by the Raman intensity ratio between 762 and 782 cm^−1^, with the greater value displaying a high proportion of the α form, as illustrated in Fig. 5 ▸. Red represents pure β polymorph with a value less than 0.700, whereas blue denotes pure α polymorph having a value greater than 1.300. Fig. 6 ▸ clearly confirms that only a partial phase transition from the β to the α polymorph occurs below 343 K (though the first transition appears slightly dominant) and the similar partial α-to-β transition above 333 K and another dominant phase transition below 333 K. These results are in agreement with the 1D VT-Raman spectral data. Together, Raman and DSC data demonstrate the presence of a two-step transition process for the reverse solid-to-solid phase transition between the α and β forms and the two transitions adopt different pathways. Moreover, the first-step phase transition (*i.e.* the transition peaks at around 323 and 298 K) appears to resemble a first-order rapid transition pathway, whereas the second transition process is strongly dependent on the presence of crystal defects, which more closely resembles the nucleation and growth mechanism.

### 3.3. Kinetics of polymorphic phase transitions   

In order to understand the mechanism of the two-stage phase transition process between the α and β forms, we quantified the transition kinetics of each stage by DSC analysis at different heating rates (Table S1 of the supporting information). The non-isothermal method of data analysis was used to calculate the apparent activation energy (Li & Brill, 2007 ▸), and the kinetics of the transition process were evaluated by Ozawa (Ozawa, 1992 ▸; Chrissafis *et al.*, 2004 ▸) and Kissinger (Starink, 2003 ▸; Kissinger, 1957 ▸) models. The derived kinetic parameters for the β-to-α phase transition were summarized in Table 1 ▸. The estimated activation energies of the first-step phase transformation for non-milled and milled polycrystalline powders are 366.7 and 427.1 kJ mol^−1^ (average values of the Ozawa and Kissinger results), respectively. The small discrepancy between the two crystal samples supports the minor influence of the milling on the first stage of phase transition. However, the difference in activation energy between the first and second stages of the phase transition is remarkable. The activation energy for the second stage of the phase transition is 761.3 kJ mol^−1^ for the milled crystal samples. The significantly larger energy barrier of the second stage of the phase transition indicates a different transition pathway from the first stage, which is consistent with the Raman data. Moreover, compared with the un-milled samples, the second stage of the phase transition of polycrystalline powders milled for 90 min was enhanced by the milling process in which crystal defects play a dominant role.

This suggests the second stage of the phase transition requires more energy to reorganize into a new crystal structure, which likely proceeds via the nucleation and growth transition pathway. The Jeziorny model, derived on the basis of the nucleation growth mechanism, was thus used for kinetic data fitting (Jeziorny, 1978 ▸; Karode *et al.*, 2018 ▸). The Jeziorny equation is given by

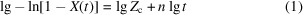

where *X*(*t*) is the relative crystallinity from the time of crystallization to time *t*, *Z*_C_ represents the kinetic rate constant of crystallization, and *n* is the Avrami index which reflects the nucleation and growth mode. When the nucleus adopts a 1D growth mode, the general *n* value is 1 or 2; an *n* value of 2 or 3 suggests a 2D growth mode; an *n* value of 3 or 4 suggests a 3D growth mode.

According to Equation (1), the relationship between lg{−ln[1 − *X*(*t*)]} and lg*t* is plotted in Figs. 6 ▸(*e*) and 6(*f*). As observed, no linear relationship between lg{−ln[1 − *X*(*t*)]} and lg*t* can be found in the first stage of the phase transition, but there is a clear linear relationship in the second stage, confirming the second stage transition to be the nucleation and growth mechanism. The derived parameters *Z*_C_ and *n* are given in Table S2. The calculated *n* values were found to be 1.4–1.5, suggesting a 1D growth mode.

### 3.4. Hot-stage polarized optical microscopy   

**Figure 7:**
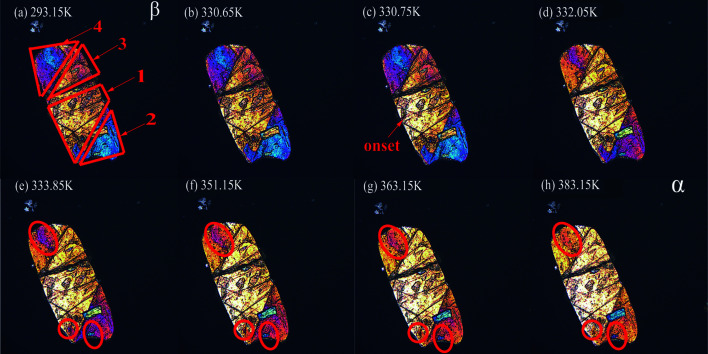
HS-POM images of the polycrystalline powders after milling for 30 min showing the change in polarization colour at different defect density zones during the heating process. (*a*) HS-POM image of the pure β form, (*b*)–(*d*) rapid first-step phase transition, (*d*)–(*h*) slow second-step phase transition [crystals in (*h*) are α form]. Defect areas are marked by red circles.

We further tracked the impact of local crystal defects on the phase transition process from β to α using a hot-stage polarized optical microscope (HS-POM). Fig. 7 ▸ visualizes this transition process, as clearly seen in the change of the polarizing colour of crystals, for polycrystalline powder samples milled for 30 min. It is evident that two transition steps exist when temperature is elevated from 293 to 383 K. The first transition step starts at 330.75 K and ends at 332.05 K, displaying rapid transition kinetics [Fig. 7 ▸(*b*)–7(*c*)] and a narrow transition period. For example, the transition process in highlighted regions 1–4 [Fig. 7 ▸(*a*)–7(*d*)] occurs almost immediately after the temperature reaches certain value. Such an observation is consistent with DSC and Raman data, supporting the low activation barrier by cooperative molecular movement. The second-step transition process proceeds mainly in local regions (outside of the first-step transition) beginning at 351 K but not finishing until 383 K. The high onset transition temperature and wide range indicate slow transition kinetics and a high transition barrier, which are in agreement with transition kinetic results (see Section 3.3). To overcome this high transition barrier, the defects region can provide a more energetically favourable place for a nucleation and growth transition pathway. Compared with collective rapid cooperative molecular motion, the phase transition process by the nucleation and growth mechanism is much slower and in a progressive way. This explains the wide range of transition temperatures observed in the second-step transition.

### 3.5. Molecular dynamics simulations   

For a better understanding of how crystal defects affect phase transitions at the molecular level, we simulated the effect of vacancy defects on the phase transition between the two polymorphs. Note that here the concept of vacancy defects, though cited directly from inorganic or ionic crystalline materials, mainly refers to the defects wherein a crystal lattice is missing at least one molecule. Phase transition simulations were performed at three hypothetical defect situations: no crystal defects, low defect density and high defect density. Crystal structures of the α and β forms both adopt hydrogen-bonded bilayer motifs (Fig. 1 ▸) where amino groups are linked to carboxyl­ate groups by a series of N—H⋯O hydrogen bonds. These bilayer units are then stacked along the [100] direction with hydro­phobic side chains lying together approximately along this direction. The hydro­phobic side chains of me­thio­nine molecules are stacked together by weak van der Waals interactions, indicating the hydrogen bonding pattern remains the same and the layer-by-layer transition occurs through the shift of the bilayer (Smets *et al.*, 2020 ▸). However, the two forms display a different stacking sequence, hydrogen bonding strength and molecular conformation. Thus, the evolution of the hydrogen bond and the movement of S atoms were tracked to visualize the mechanism of structure change during the phase transition between the two forms.

**Figure 8:**
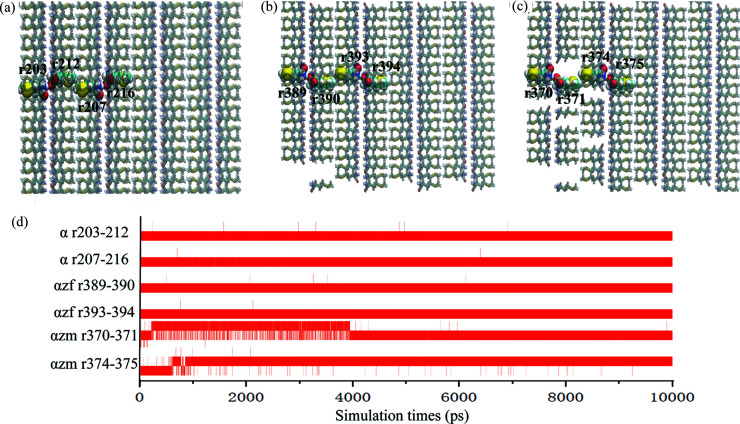
Snapshots of the initial structure of the α polymorph with (*a*) no vacancy defects, (*b*) low vacancy-defect density and (*c*) high vacancy-defect density. (*d*) Hydrogen bond lifetime under different conditions. The hydrogen bonds between molecules 203 and 212, 207 and 216 were investigated under the no defect condition; 389 and 390, 393 and 394 for low defect density; and 370 and 371, 374 and 375 for high defect density.

Fig. 8 ▸ shows the evolution of hydrogen bonding in the presence of no crystal defects, low-density defects and high-density defects as a function of simulation time in the α-to-β phase transition. The number of molecular pairs between and within layers was marked in Figs. 8 ▸(*a*)–8(*c*), whereas Fig. 8 ▸(*d*) presents the hydrogen bonding lifetime between the molecular pairs. As seen in Fig. 8 ▸(*d*), the hydrogen bonds between the bilayer units are stable when α crystals do not present any defects (αnd) or low-density of defects (αld). This suggests a phase transition in the absence of crystal defects or the presence of a low-density of defects (specifically in the defect-free region) can adopt a rapid and continuous cooperative motion pathway via the movement of hydro­phobic side chains without the breakage of strong hydrogen-bonded bilayers. In the case of crystals displaying a high defect density, the α-to-β phase transition needs to break certain hydrogen bond interactions (αhd, *e.g.* r374–375 hydrogen bond) and (re)form new hydrogen bonds (αhd, *e.g.* r370–371 hydrogen bond) when the transition is completed.

The simulation results for the reverse transformation process, from β to α, are shown in Figs. S9–S10. The similar influence of crystal defect density was observed, while we only found a partial phase transition from β to α even with a much longer simulation time (240 ns). We believe that this phase transition from β to α has the potential energy present in the structure, which blocks the crossing of the energy barrier between the two polymorphs.

**Figure 9:**
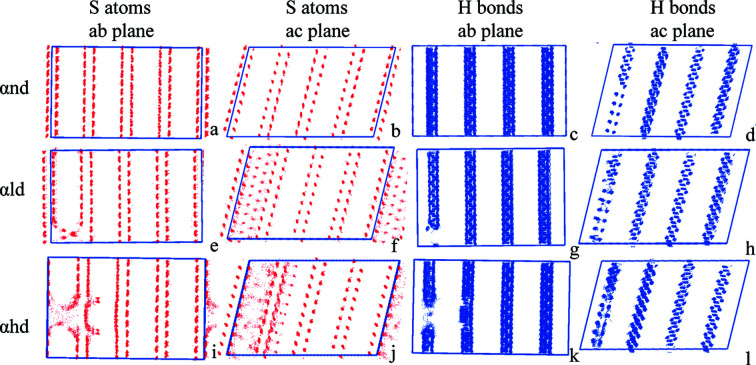
Distribution superposition of S atoms and hydrogen bonds of the α polymorph with (*a*)–(*d*) no vacancy defects, (*e*)–(*h*) low vacancy-defect density and (*i*)–(*l*) high vacancy-defect density from 0 to 10 ns. The red dot density reflects the molecular trajectory and the blue lines represent the hydrogen bond formation. The more concentrated distribution suggests a slight movement and the discrete distribution indicates a large molecular displacement.

Analyses of the distribution of S atoms and hydrogen bonds of the α polymorph superposed over a series of simulation frames also unveil the transition mechanisms in three hypothetical defect scenarios (Fig. 9 ▸). Additional inspections of the *ab* and *ac* planes show that the S atoms in the α cell display an overall shift in the absence of any crystal defects, suggesting a synchronous displacement of all molecules within the bilayer relative to the other bilayer. Furthermore, the hydrogen bonds between bilayers display no change during the phase transition. This corroborates that the transition is initiated by the hydro­phobic side chains leading to the overall movement of the bilayers. The simulation results in the presence of low crystal defect density of the α polymorph also reveals the displacement of the bilayers in the period of phase transition, which indicates a cooperative motion pathway for the transition. However, the location distribution of S atoms in the defect region becomes highly dispersed when the α polymorph has a low density of crystal defects, suggesting a larger movement of the me­thio­nine molecules. The breakage and reformation of hydrogen bonds in the hydrogen bond pattern confirms a large movement of molecules. When the α polymorph bears even higher crystal defect density, a more dispersed distribution of S atom locations and disordered distribution of hydrogen bonds in the defect region can be observed, demonstrating the large movement of me­thio­nine molecules. These large movements cannot be considered as a cooperative movement and are more likely to be compensated by the nucleation and growth pathway in the crystal phase.

### 3.6. Defect-induced phase transition pathways between polymorphs   

Previously, Smets *et al.* (2016 ▸) suggested that cooperative motion may play an important role in the solid-state phase transition of dl-me­thio­nine. In this study, we found that the phase transition of the crystals of lower defect density display a high onset transition temperature, which agrees well with their fast transition rate. However, for the crystals with high defect density (*e.g.* crystals generated by milling process), their phase transitions follow a two-step transition process where the transition adopts cooperative motion in the defect-free region, and then adopts a subsequent nucleation and growth pathway in the high defect region.

By employing DSC and *in situ* Raman spectroscopy, we found that the transition of dl-me­thio­nine polycrystalline powders includes two steps, and the extent of the second-step phase transition increases as milling time increases. The DSC curves of milled polycrystalline powders show a wider second endothermic peak than the first one, which indicates the slower rate of the second-step phase transition. Indeed, the activation energy of the second phase transition process estimated in Section 3.3 is much greater than that of the first step which occurs by cooperative molecular motion. The good linear fit in transition kinetics by the Jeziorny model suggests that the second-step transition follows the nucleation and growth mechanism. Finally, three hypothetical defect situations were studied using MD simulation to obtain a molecular level understanding of such polymorphic transformations. Crystals without and with low defect density exhibit synchronous displacement of all molecules within the bilayer and there is no large change in the hydrogen bonding. However, crystals with a high defect density undergo a large movement of me­thio­nine molecules, and hydrogen bonds between molecules are obviously broken and (re)formed in simulation times. The process of transformation clearly differs from that of the formers. Crystal structures of both forms reveal the presence of both strong hydrogen bonding and weak van der Waal interactions between the bilayers. The phase transition by cooperative motion maintains strong hydrogen bonding between the bilayers while only needing to break the weak van der Waal interactions. Thus this transition adopts a low activation energy and displays greater advantages in dynamics to complete the double-layer displacement. However, crystals of high defect density display a disordered molecular arrangement near defect domians, leading to the (partial) molecular discontinuity in crystals. The overall movement of molecules will be hindered, and the phase transformation has to be accomplished by other pathways, *e.g.* the nucleation and growth mechanism (Anwar *et al.*, 2007 ▸).

Finally, we proposed a combined two-step mechanism for the phase transition, including the cooperative motion and nucleation and growth pathways, for dl-me­thio­nine polymorphs (Scheme 1; case I represents the phase transition occurring in the presence of a low density of crystal defects, and II denotes the transition situation where the crystals have a higher defect density.). Dependent on the defect density, the phase transition of the two polymorphs can be a one-step cooperative motion mechanism or a two-step pathway including the first cooperative motion in the defect-free region and subsequent nucleation and growth at or near local defects. The transformation begins with the displacement of a few molecules at defect sites, which leads to propagation. When the defect density is very low, the β crystal transforms through cooperative displacement, and almost the whole crystal structure transforms into the α form. As the defect density is higher, the propagation process will be divided into two steps. The first step is cooperative movement at defect-free regions (seen at a lower temperature in the DSC curve), and the remaining regions complete the phase transition by the nucleation and growth pathway in the second step (at a higher temperature). Given the presence of crystal defects commonly seen in polycrystalline powders, we speculated that the cooperative motion of the phase transition will be dominated in the single-to-single crystal phase transition whereas the combined mechanism involved in both cooperative motion and nucleation and growth applies mainly to polycrystalline powders. Although the mechanism proposed here uses only dl-me­thio­nine as a model system, we believe it may also be applied to many other solid-to-solid phase transition systems. 
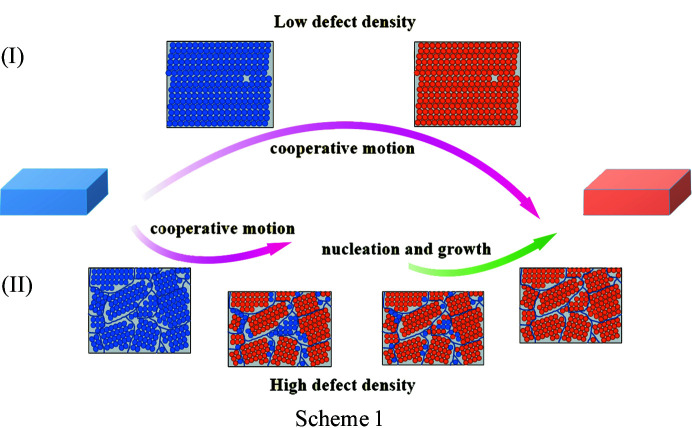


## 4. Conclusions   

This work combines thermal analyses, *in situ* Raman spectroscopy and MD simulations to explore the role of crystal defects on the solid-to-solid dl-me­thio­nine polymorphic phase transition. The polycrystalline powders were found to follow a two-step phase transition process, and the extent of the second-step transition is dependent on the density of crystal defects present in the crystals. The first-step phase transition was caused by the cooperative molecular motion mechanism, whereas the second step follows the nucleation and growth pathway at (or near) the regions of crystal defects which display a much greater energy barrier. MD simulation results further reveal that the presence of high defect density can hinder the propagation of cooperative molecular motion and cause the breakage and reformation of hydrogen-bonded bilayers when the phase transition occurs. This has to be completed by the nucleation and growth mechanism due to the extremely high energy barrier. Therefore, the solid-state phase transition between dl-me­thio­nine polymorphs proceeds initially by cooperative molecular motion outside the region of crystal defects and further completes by the nucleation and growth mechanism at (or near) the defect regions. The proposed two-step transition mechanism in this work involving both cooperative molecular motion and nucleation and growth pathways may be universal for other polymorphic crystalline materials given the ubiquitous presence of defects.

## Supplementary Material

Supporting information file. DOI: 10.1107/S2052252521004401/yc5030sup1.pdf

Video of the transition process. DOI: 10.1107/S2052252521004401/yc5030sup2.mp4

## Figures and Tables

**Table 1:** The activation energy, enthalpy, entropy and Gibbs activation energy of the β-to-α phase transition for polycrystalline powders milled for 90 min

	Kissinger		Ozawa	
Sample	*E* (kJ mol^−1^)	*R* ^2^	*E* (kJ mol^−1^)	*R* ^2^
n1	373.3	0.91	360.2	0.92
m1	435.2	0.94	419.1	0.95
m2	777.5	0.88	745.0	0.89
