# Supplementary material for: Distinct pathways of solid-to-solid phase transitions induced by defects: the case of dl-me­thio­nine
Source: IUCrJ. 2021 May 8;8(Pt 4):584–94. doi: 10.1107/S2052252521004401 (PMC8256715; doi:10.1107/S2052252521004401)
Supplement: Supplementary file 1 [file m-08-00584-sup1.pdf]

IUCrJ

Volume 8 (2021)

Supporting information for article:

Distinct Pathways of Solid-to-Solid Phase Transition Induced by Defects: the case of DL-Methionine

Genpei Shi, Si Li, Peng Shi, Junbo Gong, Mingtao Zhang and Weiwei Tang

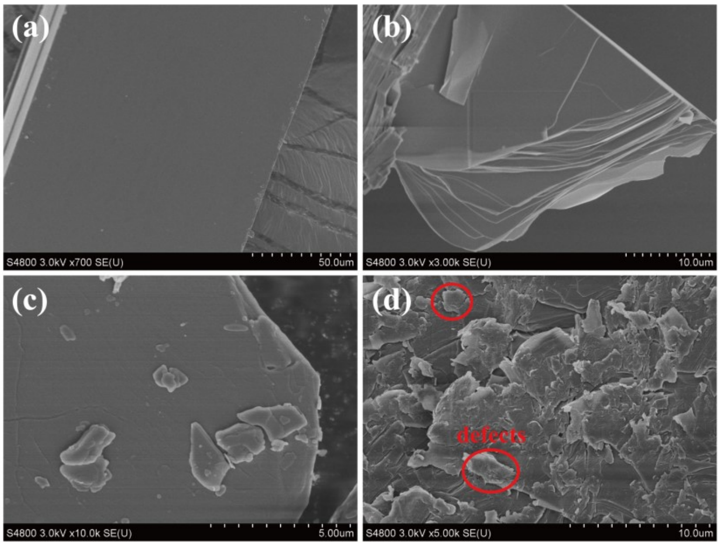

Figure S1 SEM images of Dl-methionine β form single crystals with (a) high quality and (b) low quality, as well as (c) high quality and (d) low quality polycrystalline powders.

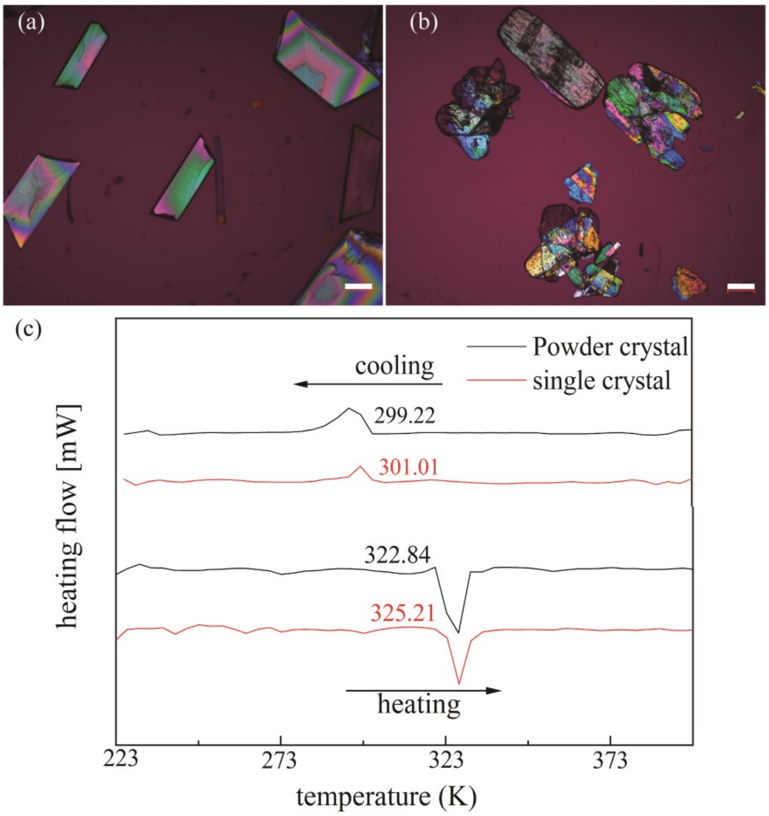

Figure S2 The polarizing microscope (PLM) images of (a) polycrystalline powders and (b) single crystals produced by cooling and slowly evaporating, respectively. The scale is 200 μm. (c) DSC thermograms of powder crystallites and single crystals.

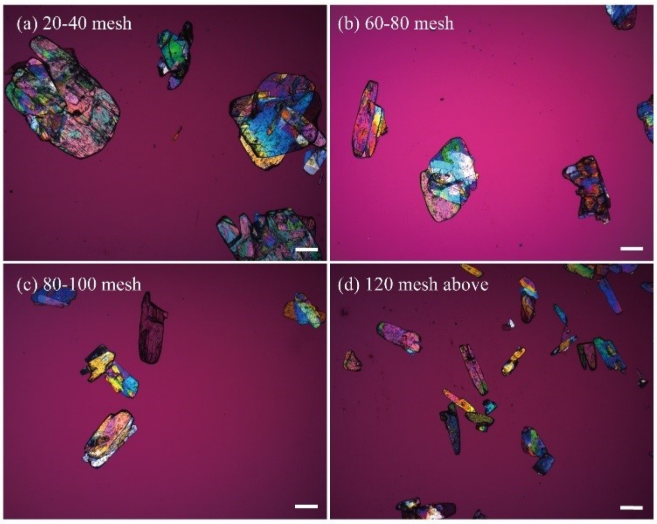

Figure S3 The PLM images of polycrystalline powders with different size for (a) 20-40 mesh(450-900 μm), (b) 60-80 mesh(180-280 μm), (c) 80-100 mesh(150-180 μm), (d) 120 mesh(<125 μm). The scale is 50 μm.

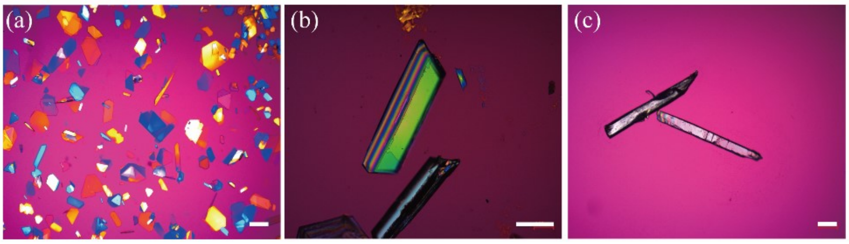

Figure S4 Single β-form DL-methionine crystals of different qualities and sizes, (a) about 200 μm, (b) about 600 μm, (c) about 1000 μm. The scale is 200 μm.

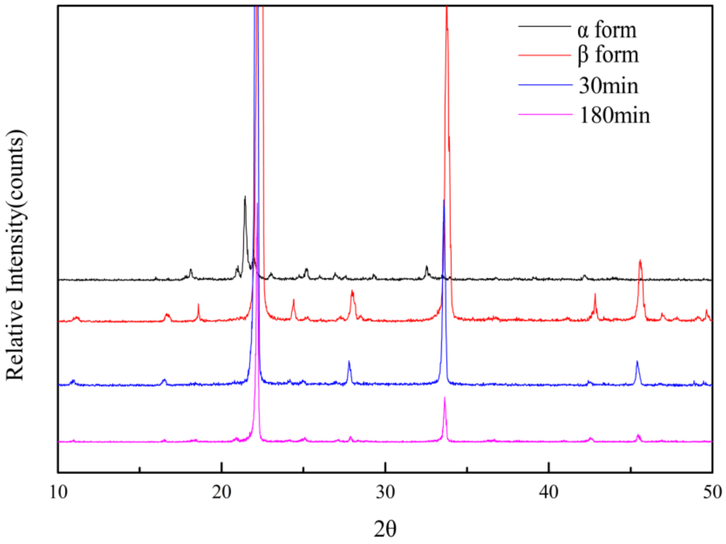

Figure S5 X-ray powder diffraction patterns of α, β forms, and β polycrystalline powders milled for 30, 180 min. the powders after milling at 10 HZ speed would still be β form.

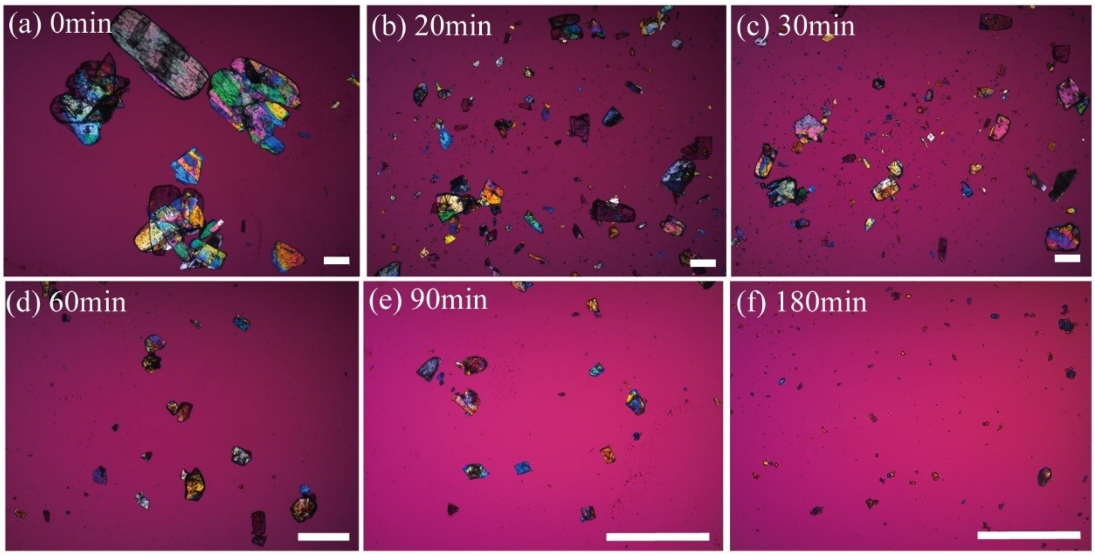

Figure S6 The PLM images of polycrystalline powders with different milling time for (a) 0 min, (b) 20 min, (c) 30 min, (d)60 min, (e) 90 min, (f)180 min. The scale is 200 μm.

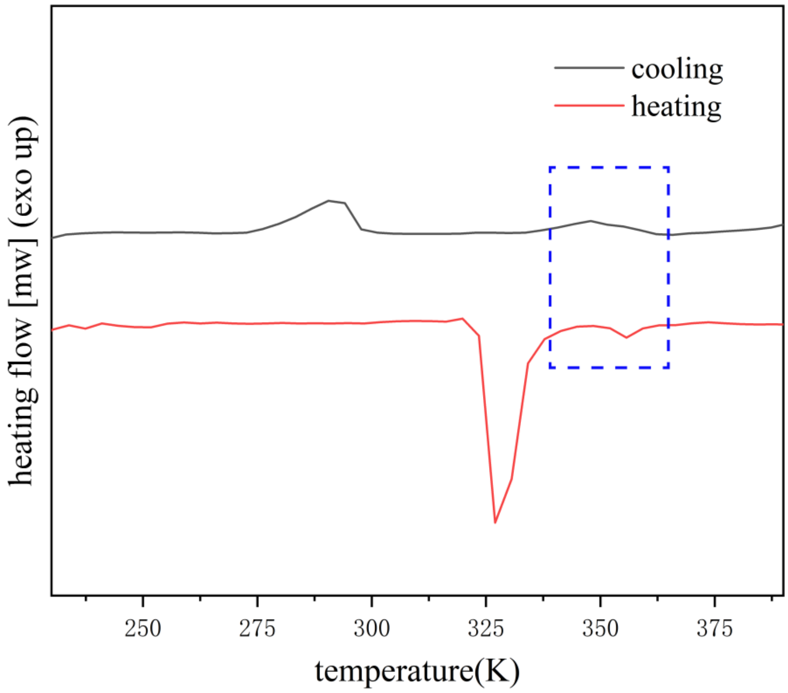

Figure S7 The DSC thermogram of low quality single crystals with small peak.

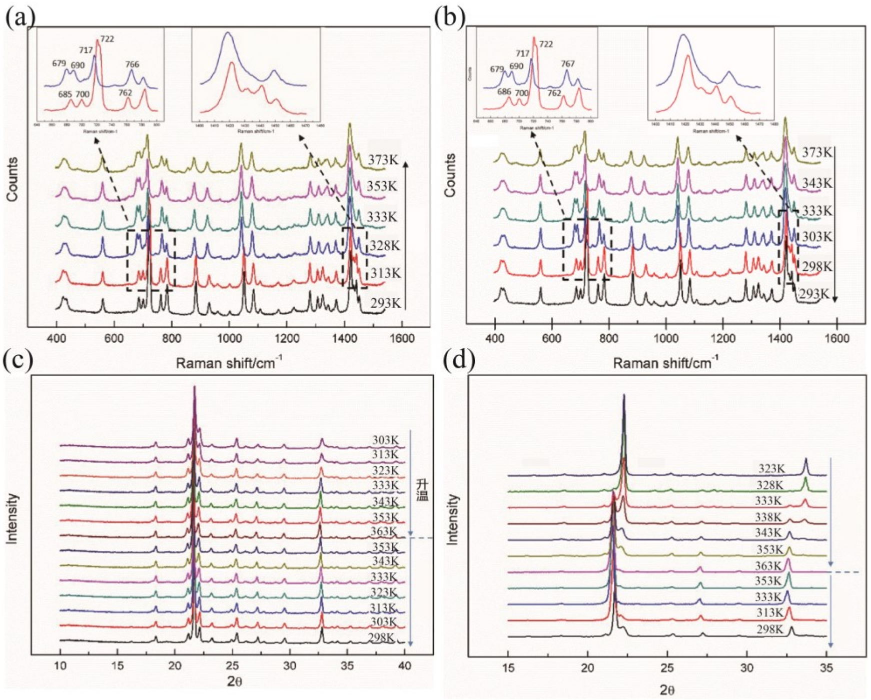

Figure S8 In situ Raman spectra of single crystal sample of DL-methionine (a) heated from 293K to 373K and (b) cooled from 373K to 293K. VT-PXRD patterns of DL-methionine polycrystalline powder samples: (c) α form heated up to 363K and then cooled to 298K; (d) β form heated up to 363K and then cooled to 298K.

Table S1 Thermal decomposition temperature of β Powders without and with milling for 90 minutes.

crystal	Heating rate（K/min）	Tonset（K）	Tp（K）	Tendset（K）
n1	2	322.63	326.19	329.39
	5	323.39	327.16	330.57
	10	323.64	327.99	333.23
	15	325.18	328.99	334.76
	20	325.7	330.1	336.94
m1	2	327.33	329.65	333.77
	5	327.82	331.02	336.69
	10	327.82	332.01	339.28
	15	328.75	332.74	340.22
	20	329.3	334.23	342.65

m2	2	346.21	355.26	357.62
	5	351.67	356.2	359.35
	10	352.32	356.36	360.47
	15	352.41	357.28	361.06
	20	353.27	358.27	362.49

n1: The first Phase Transition of nonmilled β powders; m1 and m2: the first and second phase transition of β polycrystalline powders milled for 90 min

The influence of various cooling rates under non-isothermal crystallization process on the activation energy can be described using the Kissinger equation

$$ln\left(\frac{\beta}{T_p^2}\right) = ln\left(\frac{AR}{E}\right) - \frac{E}{RT_p} \quad (1)$$

in which β represents the heating rate, T_p is the onset temperature of the transition in K, E is the activation energy of phase transition in J/mol, A is the pre-exponential factor in s^{-1}, R is the general gas constant ($8.314 J\cdot mol^{-1}\cdot K^{-1}$).

The isoconversional method of Flynn-Wall- Ozawa (FWO) is in fact a “model free” method which involves measuring the temperatures corresponding to fixed values of α from experiments at different heating rates β.

$$lg\beta = lg(\frac{AE}{RG(\alpha)}) - 0.4567\frac{E}{RT_p} - 2.315 \quad (2)$$

where G(α) is a mechanism-related function. According to Ozawa and Kissinger model, the activation energy of phase transition can be calculated by plotting lgβ (or $\ln(\beta/T_p^2)$) against $1/T_p$. Figure 6a, b show DSC curves of β form polycrystalline powders after milled 0 min and 90 min at the heating rates of 2, 5, 10, 15, and 20 K/min. As seen, the onset temperature of transition increases with the rise of the heating rate while the peak width becomes broad. By plotting lgβ against $1/T_p$, a good linear relationship can be received (Figure 6c, 6d). The Kissinger model gives the best fitting results.

Table S2 The Z_c, n values of β polycrystalline powders milled for 90 min at different heating rates

Heating rate	B /K/min	2	5	10	15	20
	Z_c /min^{-n}	0.0195	0.0930	0.2063	0.6250	1.0717
The second phase transition	n	1.48	1.48	1.50	1.39	1.52
	R^2	0.96	0.98	0.98	0.94	0.95

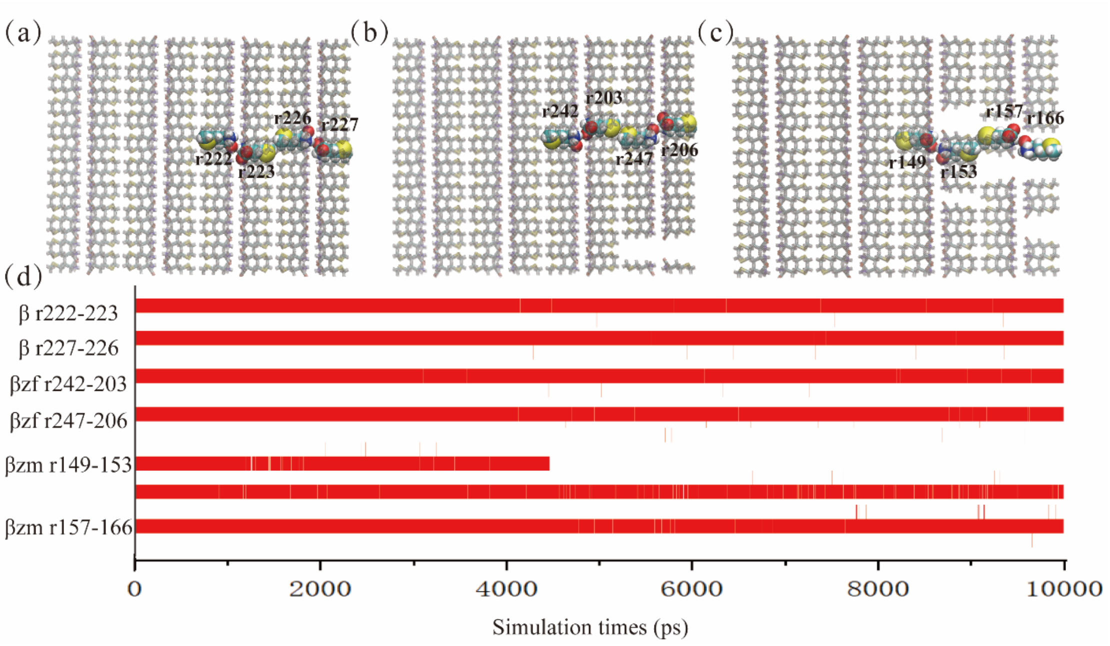

Figure S9 The snapshots of β form initial structure with (a) no vacancy defects, (b) low vacancy defect density and (c) high vacancy defect density. (d) The hydrogen bond lifetime under different conditions. The hydrogen bonds between molecules No. 222 and 223, No. 227 and 226 were investigated in the condition of no defect, while No. 242 and 203, No.247 and 206 for low defect density, and No. 149 and 153, No. 157 and 166 for high defect density.

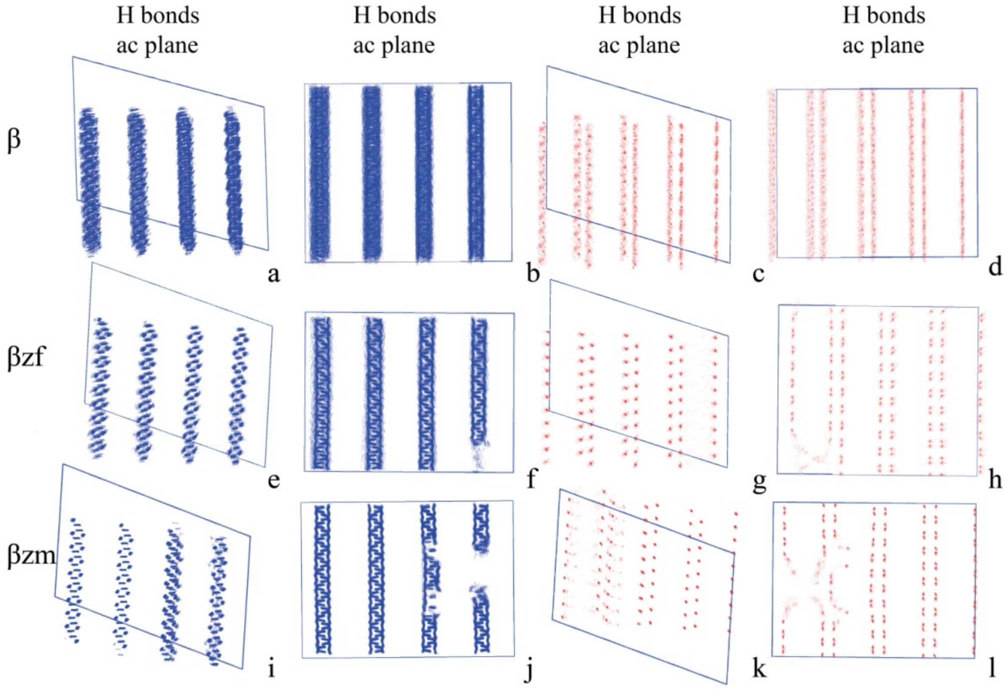

Figure S10 Distribution superposition of S atoms and hydrogen bonds of β polymorph with (a-d) no vacancy defects, (e-h) a small number of vacancy defects and (i-l) a large number of vacancy defects from 0 ns to 10 ns. The red dot density reflects the molecular trajectory and the blue line

reflects the hydrogen bonds formation. The more concentrated distribution means a slight movement while discrete distribution means a large molecular displacement.
